# A rare paediatric case with overlapping clinical features of epidermodysplasia verruciformis and pyoderma gangrenosum with hepatosplenomegaly

**DOI:** 10.1093/skinhd/vzae003

**Published:** 2025-01-20

**Authors:** Saud Azhar, Palwasha Khan, Fahad Faizullah

**Affiliations:** Dermatology Unit, Hayatabad Medical Complex, Peshawar, Khyber Pukhtunkhwa, Pakistan; Dermatology Unit, Hayatabad Medical Complex, Peshawar, Khyber Pukhtunkhwa, Pakistan; Dermatology Unit, Hayatabad Medical Complex, Peshawar, Khyber Pukhtunkhwa, Pakistan

## Abstract

Pyoderma gangrenosum (PG) is a rare neutrophilic dermatosis that typically manifests as painful ulcers, usually on the lower limbs, and is usually secondary to other conditions, for example inflammatory bowel disease and rheumatoid arthritis. Epidermodysplasia verruciformis (EDV) is an inherited or acquired primary immunodeficiency disorder linked to human papillomavirus susceptibility and an increased risk of squamous cell carcinoma. Occurrence of these conditions together has not been reported before. A 10-year-old boy presented with an extraordinary combination of clinical features consistent with PG and EDV. This unique presentation showcased recurrent nonhealing painful ulcers on the legs and body along with cribriform scarring for the last 8 years, which worsened over the last 1 year. Punch biopsy corroborated the diagnosis of PG. There were no signs and symptoms of other systemic diseases. Hypo- and hyperpigmented papules were spread all over the body with pityriasis versicolor-like macules on the forehead, neck and dorsa of the hands along with a positive family history in his younger brother, confirming the diagnosis of inherited EDV. The patient also had hepatosplenomegaly, for which paediatric workup was suggested. Treatment involved infection control, corticosteroid therapy and wound care, which led to a rapid healing response in the ulcers. This exceptional case sparks the suspicion of a novel syndrome due to its combination of EDV and PG, enhancing existing knowledge of the disease presentation.

## Teaser

A boy presented with a rare combination of pyoderma gangrenosum and epidermodysplasia verruciformis in an unprecedented co-occurrence.

**What is already known about this topic?**

- Pyoderma gangrenosum (PG) can present secondary to systemic inflammatory diseases, immunodeficiency disorders and as syndromes.
- Treatment for epidermodysplasia verruciformis (EDV) is not well established but includes immunomodulators and antimetabolites in combination with systemic steroids which is costly and long-term side effects and efficacy are unknown.

**What does this study add?**

- PG and EDV have not been known to coexist up until now.
- Hepatosplenomegaly was present concomitantly which is an atypical presentation, not reported before.
- Steroid monotherapy proved useful as first line of treatment for this condition with marked improvement.
- This spectrum of conditions namely PG, EDV and hepatosplenomegaly gives rise to the suspicion of a new syndrome and hopes to aid future diagnosis of such cases.

Pyoderma gangrenosum (PG) is a rare inflammatory disorder of the skin classically characterized by painful papules or pustules that progress to form large ulcers. It is a neutrophilic dermatosis and mostly occurs secondary to another disorder but may present on its own. The exact cause of PG is not well understood. The worldwide incidence of PG is estimated to be around 3–10 cases per million population per year.^1^

Epidermodysplasia verruciformis (EDV) is an autosomal recessive genetic disorder falling under primary immunodeficiencies. According to a systematic review conducted by Imahorn *et al.*, a total of about 500 cases have been reported worldwide since EDV was discovered.^2^ It is characterized by flat, wart-like, hypo- or hyperpigmented papules that may coalesce as scaly patches or plaques with irregular borders.^3^ The diagnosis of EDV is usually clinical.

To diagnose and manage PG effectively, it is essential to consider the comorbid conditions that can accompany it. Here we report a 10-year-old child from Pakistan with clinically diagnosed EDV, PG and hepatosplenomegaly. It highlights the importance of detailed examination of patients and picking up minor signs and symptoms that can otherwise be neglected when present concomitantly with other major clinical features. This makes this case noteworthy for clinicians and uncovers yet another unusual presentation of PG.

## Case report

A 10-year-old boy presented with an 8-year history of multiple, recurrent nonhealing painful ulcers. When he was 2 years old, the first lesion that appeared was an erythematous papule on his left flank. This lesion increased in size to form a bulla, then ruptured to form an ulcerated plaque within a few days. Mucosal surfaces were spared. Over the next 5–6 years the lesions spread to the face, buttocks and upper and lower limbs. The ulcerated plaques healed with local treatment and left pigmented atrophic scars. No specific treatment was received for this condition. The patient also reported a history of weight loss, loss of teeth without any obvious reason, undocumented fever and failure to thrive. Other systemic diseases, such as arthritis and inflammatory bowel disease, were ruled out.

The patient’s parents were second cousins and he had three younger sisters who succumbed to undiagnosed conditions marked by recurrent chest infections, failure to thrive and abdominal distension. His younger brother also had hypopigmented flat-top papules on the dorsa of both hands and his face, and multiple irregularly shaped hypopigmented patches on his trunk.

### Physical examination

**Figure 1:**
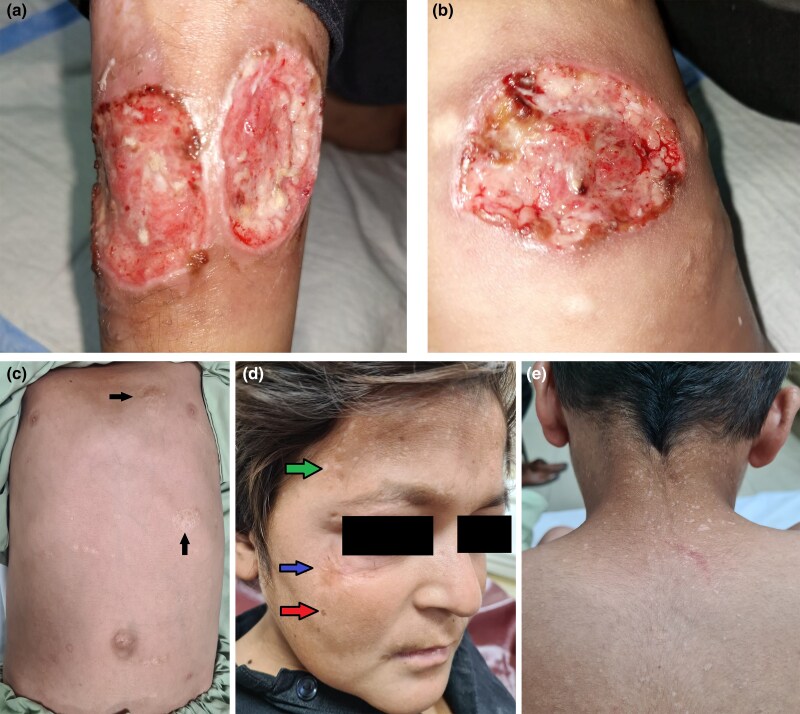
Images taken on the first day of admission. (a) Right leg showing two large ulcers on the shin. (b) One large ulcer on the left knee and hypopigmented atrophic scars around it. (c) Cribriform/cigarette paper-like scars on the abdomen. (d) Blue arrow shows hypertrophic scar under the right eye, red arrow shows seborrhoeic keratosis-like lesion and brownish macule, and green arrow shows numerous hypopigmented macules/patches (pityriasis versicolor-like) on the forehead. (e) Similar lesions were found on the dorsa of the hands and back of the neck.

The patient weighed 27 kg. On cutaneous examination, we found five ulcerated plaques of variable sizes and shapes bilaterally on the lower limbs, which were very tender. On the right leg, the largest one measured 6 × 5 cm and the other one measured 5 × 4.5 cm (Figure 1a) on the anterior aspect of the shin. On the left leg the largest one measured 6 × 5 cm (Figure 1b). The lesions were circular in shape with well-defined margins and down-sloping, violaceous borders. The base of the ulcers had granulation tissue. Apart from these, all other types of lesions and scars on the body are shown in Figure 1(c–e). On systemic examination, four permanent mandibular teeth, including incisors and canines, were absent and there was nontender abdominal ­distension.

### Investigations

A thorough work-up of the patient was done to rule out any systemic disease. The patient’s initial laboratory reports showed iron deficiency anaemia with haemoglobin (Hb) of 10.8 g dL^−1^, mean corpuscular volume 70.6 fL and serum ferritin of 27.6 ng mL^−1^. There was neutrophilic leucocytosis and reactionary thrombocytosis. All the major laboratory reports, including liver function tests, are shown in Table 1.

**Table 1:** Laboratory reports

Test	Normal range^a^	5 September 2023	9 August 2023	28 July 2023	17 July 2023
Total bilirubin (mg dL^−1^)	0.1–1.0	0.53	_	0.32	0.43
ALT (U L^−1^)	10–50	29.1	_	24.8	12.3
ALP (U L^−1^)	<300	225	_	127	190
WBC (×10^3^ cells µL^−1^)	4–11	18.8	15.63	26.5	21
Neutrophils (%)	40–75	63.8	82.8	85.7	75
Lymphocytes (%)	20–45	29.5	14	9.23	17.4
Hb (g dL^−1^)	11.5–17.5	12.1	10.9	10.8	10.2
Platelets (×10^3^ cells µL^−1^)	150–450	416	361	317	478
ESR (mm/1st hour)	0–15	40	_	_	85

Serial blood test results of the patient from the date of admission to the date of follow-up. ALP, alkaline phosphatase; ALT, alanine aminotransferase; ESR, erythrocyte sedimentation rate; Hb, haemoglobin; WBC, white blood cells. ^a^Normal laboratory values were taken from the Department of Pathology, Hayatabad Medical Complex, Peshawar, KPK, Pakistan.

Wound swab culture isolated *Pseudomonas aeruginosa*. Tests for tuberculosis and virology were negative. Urinalysis and chest X-ray were unremarkable.

On abdominal ultrasound, hepatosplenomegaly was observed; the liver and spleen measured 14.0 and 12.7 cm, respectively, both with normal parenchymal echogenicity.

A punch biopsy from the edge of one of the ulcers showed ulceration and dense acute and chronic inflammation extending into the fat, favouring PG. There was no evidence of vasculitis, granuloma formation, Leishman–Donovan bodies, atypical lymphoid granuloma or malignancy. Postbiopsy wound pathergy was performed, which turned out to be ­negative.

### Diagnosis

Because of the clinical features of plain wart-like papules, hypo- and hyperpigmented patches and a positive family history of his brother having the same skin lesions, a diagnosis of inherited EDV was ascertained. These characteristic lesions of EDV were consistent with other cases around the world.^4^

A differential diagnosis of ecthyma gangrenosum was under consideration as well after isolating *P. aeruginosa*, but was ruled out as there was no response to ulcer healing on intravenous (IV) antibiotic. Furthermore, based on the rapidly progressing ulcers, cribriform scarring, chronic and recurring history of ulcers, characteristic morphology of ulcers, histopathological findings and rapid response to steroid therapy, the diagnostic criteria of PG were fulfilled after excluding all other causes of similar lesions.

In our case, hepatosplenomegaly was accompanied by a history of loss of teeth, developmental regression and a similar family history of the three siblings, along with ulcer formations, which, after ruling out common causes, was suspected to be a potential lysosomal storage disease, more specifically Niemann–Pick type B or milder forms (type I and II) of mucopolysaccharidosis, as these signs were found in patients with lysosomal storage diseases.^5^ However, upon follow-up ultrasound of the patient, the liver had regressed in size, hence a liver biopsy was not indicated, and the diagnosis of lysosomal storage disease was ruled out after paediatric consultation.

### Management

Intravenous dexamethasone 1 mL (4 mg) once daily was commenced after lack of clinical response to IV tazocin, along with saline soaks for wound care. This led to the lesions becoming nontender and starting to heal as they responded within a week of the starting dose. Wounds were measured weekly using ulcer charting. The largest ulcer before discharge measured 3.8 × 2 cm.

IV dexamethasone was switched to 25 mg oral prednisolone after 18 days. For symptomatic treatment zinc oxide paste, sucralfate, dietary supplements and oxoferin drops were given. The patient improved over the course of 25 days and was discharged. He was prescribed to continue the tapering oral steroid therapy for 20 days and then come in for follow-up.

### Follow-up and outcome

**Figure 2:**
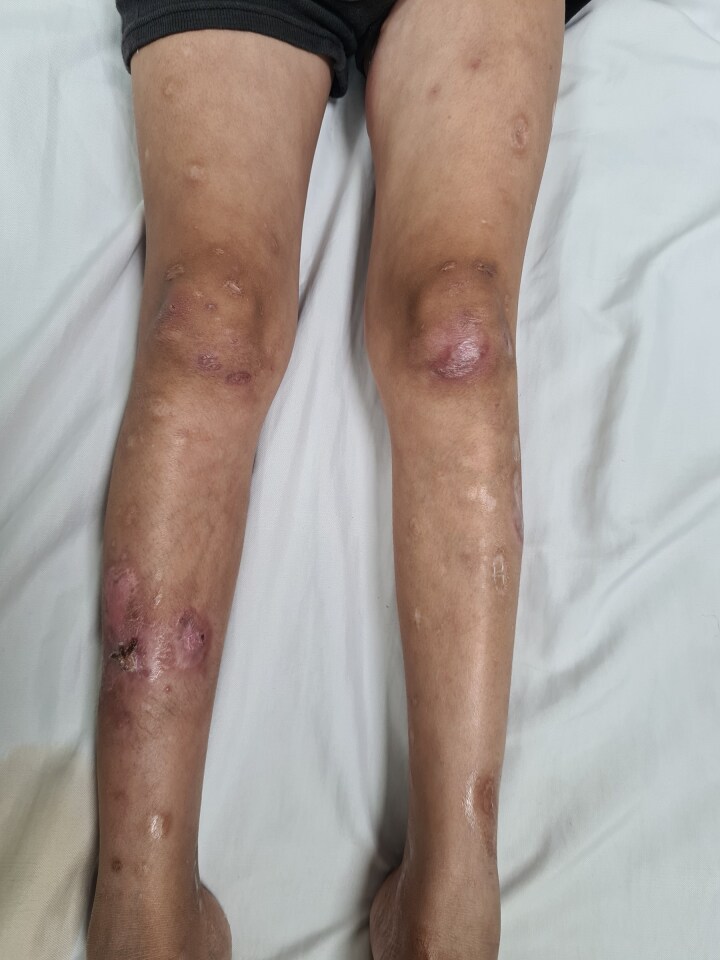
Follow-up image showing healed ulcers on the legs leaving behind atrophic scars.

The patient returned for a follow-up after 25 days looking healthy with his weight increased to 33 kg. He had no new lesions and the previous ones had resolved almost completely, as depicted in Figure 2. All the investigations were repeated, as shown in Table 1. Ultrasound of the abdomen was repeated, which showed a reduced liver but still a bulky spleen.

### Patient counselling

The patient and his father were previously perplexed by their undiagnosed condition despite numerous consultations with the local doctors. Subsequently, our medical team accurately diagnosed and informed them of this rare, lifelong autoimmune disease with a remitting relapsing pattern of ulcers that could be managed with a good outcome, but the long-term prognosis and life expectancy were unpredictable. They received this information with optimism and expressed contentment upon receiving a diagnosis, as it provided answers to many lingering questions. The pair willingly supported the medical team's decision to document the case for global benefit and expressed gratitude for the positive outcome, with the child now leading a healthy and active life with no new wounds.

## Discussion

According to a systematic review published in 2017, commonly reported underlying diseases in paediatric PG are inflammatory bowel diseases, haematological disorders, vasculitis, immune deficiencies and pyogenic arthritis. More than half of the cases occurred with no underlying disease.^6^ PG is known to present as other syndromes such, as pyoderma gangrenosum–acne–hidradenitis suppurativa (PASH) and pyogenic arthritis–pyoderma gangrenosum–acne (PAPA) syndrome.^7^

In rare instances, PG has appeared with primary immunodeficiency disorders other than EDV, for example IgA deficiency,^8^ chronic granulomatous disease^9^ and common variable immunodeficiency.^10^ Apart from this, PG has also been associated with hidradenitis suppurativa and mastitis.^11^ From Pakistan only two cases have been reported, showing PG associations with Covid-19 and thyroid disease.^12,13^

Very few cases of inherited EDV have been reported. Associated diseases reported with EDV are neurofibromatosis type 1, thymoma, Hansen disease, herpes simplex labialis and eccrine poroma, coeliac disease and porokeratosis of Mibelli, systemic lupus erythematosus and angiokeratoma of Fordyce, tinea cruris and asthma.^14^

It cannot be ascertained whether this is a mere coexistence of the two diseases or there is some underlying association. After meticulous literature review, it was observed that PG has emerged with a few other primary immunodeficiency disorders, as mentioned above. Hence it can be said that although the diseases are not directly linked, they may have an indirect association. Further case series are needed to establish a definite association. Having said that, no case has been documented worldwide with such a spectrum of conditions, namely EDV, PG and hepatosplenomegaly.

## Data Availability

All data generated or analysed during this study are included in this article. Further enquiries can be directed to the corresponding author.

## References

[1] Monari P, Moro R, Motolese A et al Epidemiology of pyoderma gangrenosum: results from an Italian prospective multicentre study. Int Wound J 2018; 15:875–79. 29877043 10.1111/iwj.12939 PMC7949684

[2] Imahorn E, Yuksel Z, Spoerri I et al Novel TMC8 splice site mutation in epidermodysplasia verruciformis and review of HPV infections in patients with the disease. J Eur Acad Dermatol Venereol 2017; 31:1722–26. 28646613 10.1111/jdv.14431

[3] Leiding JW, Holland SM. Warts and all: human papillomavirus in primary immunodeficiencies. J Allergy Clin Immunol 2012; 130:1030–48. 23036745 10.1016/j.jaci.2012.07.049 PMC3517887

[4] Vora RV, Kota RS, Singhal RR, Gandhi SS. A sporadic case of epidermodysplasia verruciformis in a young boy. Indian J Paediatr Dermatol 2017; 18:335–37.

[5] Sun A. Lysosomal storage disease overview. Ann Transl Med 2018; 6:476. 30740407 10.21037/atm.2018.11.39 PMC6331358

[6] Kechichian E, Haber R, Mourad N et al Pediatric pyoderma gangrenosum: a systematic review and update. Int J Dermatol 2017; 56:486–95. 28233293 10.1111/ijd.13584

[7] Huang J, Tsang LS, Shi W, Li J. Pyoderma gangrenosum, acne, and hidradenitis suppurativa syndrome: a case report and literature review. Front Med (Lausanne) 2022; 9:856786. 35402426 10.3389/fmed.2022.856786 PMC8987973

[8] Demirel S, Shetty M, Patel M, Mahmood K. First presentation of pyoderma gangrenosum in a patient with partial immunoglobulin A deficiency. JRSM Open 2022; 13:20542704221086386. 10.1177/20542704221086386 PMC9019357 35464105

[9] Tharwat S, Ahmed AA. Pyoderma gangrenosum and chronic granulomatous disease treated with adalimumab: case-based review. Egypt Rheumatol 2021; 43:189–92.

[10] Simsek O, Ulusan K, Orhan A et al Pyoderma gangrenosum with common variable immunodeficiency. Wounds 2015; 27:​129–33. 25965182

[11] Breznik V, Marko PB. A case of overlapping clinical features of idiopathic granulomatous mastitis, hidradenitis suppurativa, and pyoderma gangrenosum successfully treated with adalimumab. Case Rep Dermatol 2022; 14:98–106. 35702373 10.1159/000523801 PMC9149353

[12] Kubaisi T . A neglected pyoderma gangrenosum following COVID-19: a case report. J Pak Assoc Dermatol 2023; 33:345–49.

[13] Mansoor M, Butt G, Asad A et al Pyoderma gangrenosum – a case report. J Dermat Cosmetol 2020; 4:59–60.

[14] Bhutoria B, Shome K, Ghosh S et al Lewandowsky and Lutz dysplasia: report of two cases in a family. Indian J Dermatol 2011; 56:190–93. 21716545 10.4103/0019-5154.80414 PMC3108519

